# Cassava (*Manihot esculenta*) Slow Anion Channel (*MeSLAH4*) Gene Overexpression Enhances Nitrogen Assimilation, Growth, and Yield in Rice

**DOI:** 10.3389/fpls.2022.932947

**Published:** 2022-06-27

**Authors:** Linhu Song, Xingmei Wang, Liangping Zou, Zakaria Prodhan, Jiaheng Yang, Jianping Yang, Li Ji, Guanhui Li, Runcong Zhang, Changyu Wang, Shi Li, Yan Zhang, Xiang Ji, Xu Zheng, Wanchen Li, Zhiyong Zhang

**Affiliations:** ^1^State Key Laboratory of Wheat and Maize Crop Science and Center for Crop Genome Engineering, College of Agronomy, Henan Agricultural University, Zhengzhou, China; ^2^College of Life Sciences, Neijiang Normal University, Neijiang, China; ^3^Institute of Tropical Bioscience and Biotechnology, Chinese Academy of Tropical Agricultural Sciences, Haikou, China; ^4^Key Laboratory of Biology and Genetic Improvement of Maize in Southwest Region, Ministry of Agriculture, Maize Research Institute, Sichuan Agricultural University, Chengdu, China

**Keywords:** cassava, slow anion channel, transgenic rice, nitrogen use efficiency, root phenotype

## Abstract

Nitrogen is one of the most important nutrient elements required for plant growth and development, which is also immensely related to the efficient use of nitrogen by crop plants. Therefore, plants evolved sophisticated mechanisms and anion channels to extract inorganic nitrogen (nitrate) from the soil or nutrient solutions, assimilate, and recycle the organic nitrogen. Hence, developing crop plants with a greater capability of using nitrogen efficiently is the fundamental research objective for attaining better agricultural productivity and environmental sustainability. In this context, an in-depth investigation has been conducted into the cassava slow type anion channels (*SLAHs*) gene family, including genome-wide expression analysis, phylogenetic relationships with other related organisms, chromosome localization, and functional analysis. A potential and nitrogen-responsive gene of cassava (*MeSLAH4*) was identified and selected for overexpression (OE) analysis in rice, which increased the grain yield and root growth related performance. The morpho-physiological response of OE lines was better under low nitrogen (0.01 mm NH_4_NO_3_) conditions compared to the wild type (WT) and OE lines under normal nitrogen (0.5 mm NH_4_NO_3_) conditions. The relative expression of the *MeSLAH4* gene was higher (about 80-fold) in the OE line than in the wild type. The accumulation and flux assay showed higher accumulation of ${\mathrm{NO}}_{3}^{-}$ and more expansion of root cells and grain dimension of OE lines compared to the wild type plants. The results of this experiment demonstrated that the *MeSLAH4* gene may play a vital role in enhancing the efficient use of nitrogen in rice, which could be utilized for high-yielding crop production.

## Introduction

Nitrogen (N) is one of the most important macronutrients and plays a significant role in the photosynthesis, growth, development, and reproduction of plants. Nitrogen is an essential component element of many enzymes that control and direct many biochemical reactions in plants (Kraiser et al., 2011; Li et al., 2020). Therefore, N availability and utilization are the key factors for adequate biomass accumulation, proper crop growth, yield, and productivity. Plants can absorb N from various sources in the form of organic nitrogen compounds, nitrate ( ${\mathrm{NO}}_{3}^{-}$ ) and ammonium ( ${\mathrm{NH}}_{4}^{+}$ ; Vidal et al., 2020). The efficient use of N by crop plants involves several steps, including uptake, assimilation, translocation, and, when the plant ages, recycling and remobilization (Masclaux-Daubresse et al., 2010). Furthermore, N plays an important signaling role in plant growth and metabolism, such as breaking seed dormancy, controlling lateral root and leaf development, regulating blooming time, and activating associated genes (Ho and Tsay, 2010; Hachiya and Sakakibara, 2017).

Plants have evolved a sophisticated mechanism for up-taking, allocation, and storage of inorganic and organic N, including low-affinity transport systems (LATS), which operate at high nutrient concentrations (> 1 mm), and high-affinity transport systems (HATS) that predominate in the micromolar range (Wang et al., 1993; Kraiser et al., 2011). There are four gene families, including *Nitrate Transporter 1*/*Peptide Transporter* (*NRT1*/*PTR*, *NPF*), *NRT2*, *Chloride Channel* (*CLC*), and *Slow Anion Channel* (*SLAC1*/*SLAH*), involved in the nitrate transport system (Krapp et al., 2014; O'Brien et al., 2016). Among these subfamilies, the *SLAC*/*SLAH* members play an important role in anion transport, stress signaling, growth and development, and hormonal response in plants (Vahisalu et al., 2008; Nan et al., 2021).

Slow anion channel proteins, particularly those implicated in nitrate absorption and transport, are the subject of an increasing amount of research. Members of the *SLAC*/*SLAH* family have been found and researched in a variety of plants, including *Arabidopsis* (Zheng et al., 2015), rice (Sun et al., 2016), maize (Qi et al., 2018), barley (Liu et al., 2014), tobacco (Kurusu et al., 2013), poplar (Jaborsky et al., 2016), pear (Chen et al., 2019a), and *Brassica napus* (Nan et al., 2021). A total of five *SLAC*/*SLAH* genes were identified in *Arabidopsis* (Vahisalu et al., 2008), 23 genes in *B. napus* (Nan et al., 2021), and 9 genes in rice (Sun et al., 2016). Differential expression and assembly of *SLAH1*/*SLAH3* anion channel subunits are used by plants to regulate the transport of ${\mathrm{NO}}_{3}^{-}$ and Cl^−^ between the root and shoot, where the *AtSLAH1* gene is co-localized with *AtSLAH3* (Cubero-Font et al., 2016). The *SLAC1* anion channel, as well as its homologs, *SLAH3* and *SLAH2*, have been functionally characterized in *Arabidopsis* and *Xenopus* oocytes (Negi et al., 2008; Maierhofer et al., 2014a). The *SLAC1* gene is mostly found in guard cells and is phosphorylated by the *Open stomata 1* (*OST1*) kinase, causing anion efflux from guard cells, which mediates stomatal closure and increases drought tolerance (Vahisalu et al., 2010; Geiger et al., 2011). The interaction of *AtSLAC1* and *AtSLAH3* with several kinase phosphatases is linked to water stress signals (Brandt et al., 2012). The SLAH3 protein is phosphorylated by calcium-dependent protein kinases such as CPK2 and CPK20, which regulate pollen tube formation *via* regulating *SLAC*/*SLAH* expression in *Arabidopsis* (Gutermuth et al., 2013). The *SLAH2* gene, which is the most similar protein to *SLAH3*, absorbs only nitrate, unlike other *SLAC*/*SLAH* members, which absorb both nitrate and chloride (Maierhofer et al., 2014b). The *PbrSLAH3* gene is localized in the plasma membrane without expression in flowers, and has a strong selective absorption for nitrate and no permeability to chlorine (Chen et al., 2019b). Nevertheless, the *PttSLAH3* gene of poplar is not activated by protein kinase phosphorylation to absorb nitrate and chloride ions (Jaborsky et al., 2016). The *AtSLAH4* gene, which is phylogenetically linked to *AtSLAH1*, has a similar expression pattern as *AtSLAH3*, but is greater toward the root tip (Zheng et al., 2015). However, research on the *SLAH4* gene is very limited and molecular, physiological, and functional studies have not been carried out completely, which makes *SLAH4* a promising candidate gene for plant genetic engineering and biotechnological investigations.

Cassava is a short-day dicot plant in the Euphorbiaceae family that is used as a food crop as well as a possible biofuel crop (Drunkler et al., 2012). Cassava is a durable and easy-to-plant tropical commercial crop with a high degree of adaptability, and it may produce a huge yield in dry and barren mountainous and hilly areas (Wang, 2002). Cassava can obtain sufficient nitrogen from the soil to fulfill its own growth and development requirements without requiring excessive nitrogen fertilizer throughout the growing phase (Jiang et al., 2016). Therefore, it is crucial to identify the key genes involved in cassava’s nitrogen-efficient utilization for the improvement and production of nitrogen-efficient germplasm resources through genetic modification in other crops, particularly in rice.

In this study, six *SLAH* genes were identified in the cassava genome and their phylogenetic relationships, chromosomal localization, and morpho-physiological characteristics have been analyzed. A potential candidate gene, *MeSLAH4*, which was localized in the plasma membrane and in the nucleus, highly expressed in the roots under low nitrate conditions, was selected for overexpression analysis. Furthermore, several parameters related to plant growth, development, and yield were evaluated to demonstrate the role of this gene in improving nitrogen use efficiently in rice. The results of these experiments suggested that overexpression of *MeSLAH4* could increase plant growth, grain dimension, root systems indices (root morphology), and yield in rice. The findings of this research would shed new light on the possibility of a genetic engineering approach of key candidate genes in nitrogen uptake and utilization.

## Materials and Methods

### Plant Materials and Growth Conditions

The cassava (*Manihot esculenta*) variety (“Huanan5”) was chosen as the wild type during this experiment. Cassava seedings were grown on half-strength Murashige and Skoog (MS) medium at 26°C with a 16 h light and 8 h dark cycle and 70% relative humidity conditions in a growth chamber. The tobacco (*Nicotiana benthamiana*) plants were also grown in a greenhouse under cycles of 16 h light and 8 h dark at 25°C. The rice (*Oryza sativa*) seedlings were grown in controlled conditions of 16 h light (30°C) and 8 h dark (28°C) photoperiods with ~70% relative humidity. For the overexpression experiment, the coding sequences of the *MeSLAH4* gene were amplified and inserted into pCAMBIA2300-35S-eGFPvector. These constructs were subsequently transferred into an *Agrobacterium* strain (EHA105) for rice transformation. For the hydroponic culture, the plants were grown in a nutrient solution containing 1.5 mm NH_4_NO_3_, 0.3 mm NaH_2_PO_4_, 0.3 mm K_2_SO_4_, 1.0 mm CaCl_2_, 1.6 mm MgSO_4_, 0.5 mm Na_2_SiO_3_, 20 μm Fe-EDTA, 18.9 μm H_3_BO_3_, 9.5 μm MnCl_2_, 0.1 μm CuSO_4_, 0.2 μm ZnSO_4_, and 0.39 mm Na_2_MoO_4_, but supplied with different N concentrations, termed as normal nitrogen NN (0.5 mm NH_4_NO_3_) and low nitrogen LN (0.01 mm NH_4_NO_3_), pH 5.5. The nutrient solution was changed every 3 days.

### Phylogenetic Analysis and Expression Pattern of Cassava *SLAH* Genes

The protein sequences of *SLAH* genes (gene name; locus identifiers) of *Arabidopsis*, including *AtSLAH1* (*AT1G12480*), *AtSLAH1* (*AT1G62280*), *AtSLAH2* (*AT4G27970*), *AtSLAH3* (*AT5G24030*) and *AtSLAH4* (*AT1G62262*), were downloaded from the TAIR database^1^ (Swarbreck et al., 2007). The SLAH protein sequences of genes in rice, such as *Os01g0623200* (*LOC_Os01g43460*), *Os01g0385400* (*LOC_Os01g28840*), *Os05g0219900* (*LOC_Os05g13320*), *Os07g0181100* (*LOC_Os07g08350*), *Os01g0226600* (*LOC_Os01g12680*), *Os04g0574700* (*LOC_Os04g48530*), *Os01g0247700* (*LOC_Os01g14520*), *Os05g0269200* (*LOC_Os05g18670*), and *Os05g0584900* (*LOC_Os05g50770*) were downloaded from the RAP_DB database.^2^ The SLAH protein sequence of *M. esculenta* (Cassava), including *MANES_05G153100* (SLAH4), *MANES_11G124900* (SLAC1 homolog 1), *MANES_14G020300* (SLAC1 homolog 3), *MANES_06G154500* (SLAC1 homolog 3), *MANES_06G154600* (SLAC1 homolog 3), and *MANES_S089100*, along with their homologues in other species such as *Zea mays* (Corn), *Triticum aestivum* (Wheat), *B. napus* (Rapeseed), *Selaginella moellendorffii* (Spikemoss) etc., were downloaded from the Phytozome database^3^ (Nan et al., 2021). The phylogenetic trees were constructed based on the SLAC/SLAH protein sequences by IQ-TREE using the Maximum Likelihood (ML) method with 1,000 replicates of bootstrap alignments. RNA-seq data and differential gene expression information were obtained from a published database.^4^

### Chromosomal Localization Analysis

The chromosomal localization information of the *SLAC*/*SLAH* genes was obtained from sequences of the cassava genome, and the MG2C^5^ was used to draw the chromosomal distribution of *MeSLAH* genes.

### Gene Structure and Conserved Motifs Analysis

The structures of the *SLAH* genes were analyzed using the Gene Structure Display Server (GSDS 2.0)^6^ by aligning the cDNA sequences with their corresponding genomic DNA sequences. Conserved motifs of the SLAH proteins were identified using the online Multiple Expectation Maximization for Motif Elicitation (MEME^7^; Bailey et al., 2006). All obtained SLAH protein sequences were analyzed against the Pfam database to verify the presence of SLAC1 domains (Supplementary Figures 2, 3). The SLAC1 domain was also detected by the SMART program (SMART).^8^ Protein sequences lacking the SLAC1 domain or having E-values of more than 1 e-6 were removed.

### Subcellular Localization of MeSLAH4 Protein

Rice protoplasts were isolated for transient transformation of the *MeSLAH4* gene (Zhang et al., 2011; Burman et al., 2020). The open reading frame (ORF) of the *MeSLAH4* gene was amplified by PCR (95°C for 5 min, then 35 cycles of 95°C for 30 s, 58°C for 30 s, and 72°C for 70 s, with a final extension at 72°C for 5 min). The PCR products (Supplementary Figure 4) were cloned into the 35S-eGFP vector in between the XhoI and HindIII sites and under the control of the cauliflower mosaic virus 35S promoter. The competent cells of *Escherichia coli* (DH5α) and *Agrobacterium* (LBA4404) were used for the transformation of recombinants. Listed primers (Supplementary Table 2) for gene cloning and vector construction and plasma membrane marker (35 s-ZmCDPK7-MCHERRY and 35S-HY5-Mcherry; Zhao et al., 2021) were used in this study. The MeSLAH4-eGFP and 35 s-ZmCDPK7-MCHERRY fusion constructs were transiently expressed in rice protoplasts using a polyethylene glycol calcium-mediated method. An empty 35S-GFP vector was served as a negative control. Transfected protoplasts were observed after 16 h of incubation by a confocal laser scanning microscope (Olympus FV3000, Tokyo, Japan).

### Measurements of Morpho-Physiological Traits in Rice

The morphological, physiological, and agronomic traits of each transgenic rice line (OE) and wild type (WT) was measured at the 2-week-seedling stage and the maturity stage. The morphological characters, including seedlings height (cm) and root length (cm), were measured using a ruler or meter stick. The grains were lined up lengthwise and widthwise along a ruler to measure grain length (mm) and grain breadth (mm), respectively, and the measurements were confirmed using an MRS-9600TFU2L (MICROTEK) grain observation instrument. The shoot weight (g. plant^−1^ FW), root weight (g. plant^−1^ FW), and grain yield of a single spikelet (g) were measured using a weighing balance. The root fork numbers (number of branches), root tip numbers, and grain numbers of a single spikelet were calculated with the eye and confirmed by capturing an image with an Epson Expression 10000XL (Epson, Japan) and counting with winRHIZO software (Li et al., 2016a). The root average diagram (mm), root surface area (cm^2^), root volume (cm^3^), and grain diameter (mm) measurements were performed using a microscope (MVX10, Olympus), and the winRHIZO scanner-based image analysis system (Regent Instruments, Montreal, QC, Canada; Sun et al., 2014). Total grain protein content (%), and grain moisture content (%) were detected using an XDS Near-Infrared Rapid Content Analyzer (Foss® Analytical, Hilleroed, Denmark; Li et al., 2014).

The chlorophyll was extracted from 0.15 g of fresh leaves at the booting stage using 95.0% ethanol. Briefly, leaves were cut into 3 mm pieces and immersed in 95.0% ethanol for 24 h in the dark at 26°C. The absorbance of the extract was measured using a spectrophotometer at A665 and A649. The chlorophyll-a content (mg. g^−1^ FW), chlorophyll-b content (mg. g^−1^ FW), and total chlorophyll content (mg. g^−1^ FW) contents were determined by the method reported by Arnon (1949). A total of 10 individuals of each transgenic line and wild type plants were assayed (Li et al., 2016b).

The activities of catalase (CAT), peroxidase (POD), and superoxide dismutase (SOD) were measured by employing 0.5 g of seedlings in 5 ml of extraction buffer containing 0.05 M phosphate buffer (Li et al., 2018). The CAT activity was determined spectrophotometrically based on the decrease in absorbance of H_2_O_2_ (extinction coefficient of 43.6 M^−1^ cm^−1^) at 240 nm for 1 min (Aebi, 1984). The POD was measured as the absorbance at 470 nm. The SOD activity was assayed by measuring the ability of the enzyme extract to inhibit the photochemical reduction of nitroblue tetrazolium (NBT; Yoshimura et al., 2000). Phenotypic measurements of the positive transgenic plants were undertaken using three independent lines at least (Li et al., 2014).

### Gene Expression Analysis Using Quantitative Real-Time PCR

Total RNA was isolated from different tissues (leaves, stems, and roots) of cassava and rice using an RNA kit (TRNzol universal reagent, TIANGEN Biotech, Beijing, China) according to the manufacturer’s instructions. The RNA was then reverse transcribed into cDNA using the oligo (dT) primers and ImProm-II reverse transcriptase (Promega). The specific primers for the *MeSLAH4* genes along with the housekeeping Actin genes were designed using the Primer Premier 5.0 software (Supplementary Table 2). The real-time PCR reactions were conducted with 2 μl of diluted cDNA, 200 nM of each primer, 2× SYBER GREEN Master Mix (Green I Master Mix, Roche) in a final volume of 20 μl of double sterile water. The thermal cycle conditions were pre-incubation at 95°C for 5 min, then 40 cycles of 95°C for 3 s, 60°C for 10 s, and 72°C for 30 s, with a final extension at 72°C for 3 min in the Light Cycler 480 (Roche, United States). The gene expression levels were calculated with the 2^−ΔΔCt^ method (Livak and Schmittgen, 2001), and the qRT-PCR assays were performed with three biological and three technical replicates.

### Nitrogen Accumulation Analysis

Fresh samples (whole plant, grain, and glume) of WT or transgenic lines were harvested at the rice mature stage (*n* = 4) and heated at 105°C for 30 min. The samples were then dried for 3 days at 75°C. Dry weights were recorded as biomass values. Total N accumulation was assessed using the Kjeldahl method in the different plant samples by multiplying the N concentration with the corresponding biomass.

### Statistical Analysis

The data from the experiments were analyzed by one-way ANOVA, Duncan’s multiple range test, and Tukey’s test at *p* < 0.05 to determine the statistically significant differences among different treatments. All the statistical evaluations were performed using SPSS version 20.0 statistical software (SPSS Inc., Chicago, IL, United States) and MS-Office 2019 software.

## Results

### Phylogenetic and Expression Pattern Analysis of SLAH Genes

**Figure 1:**
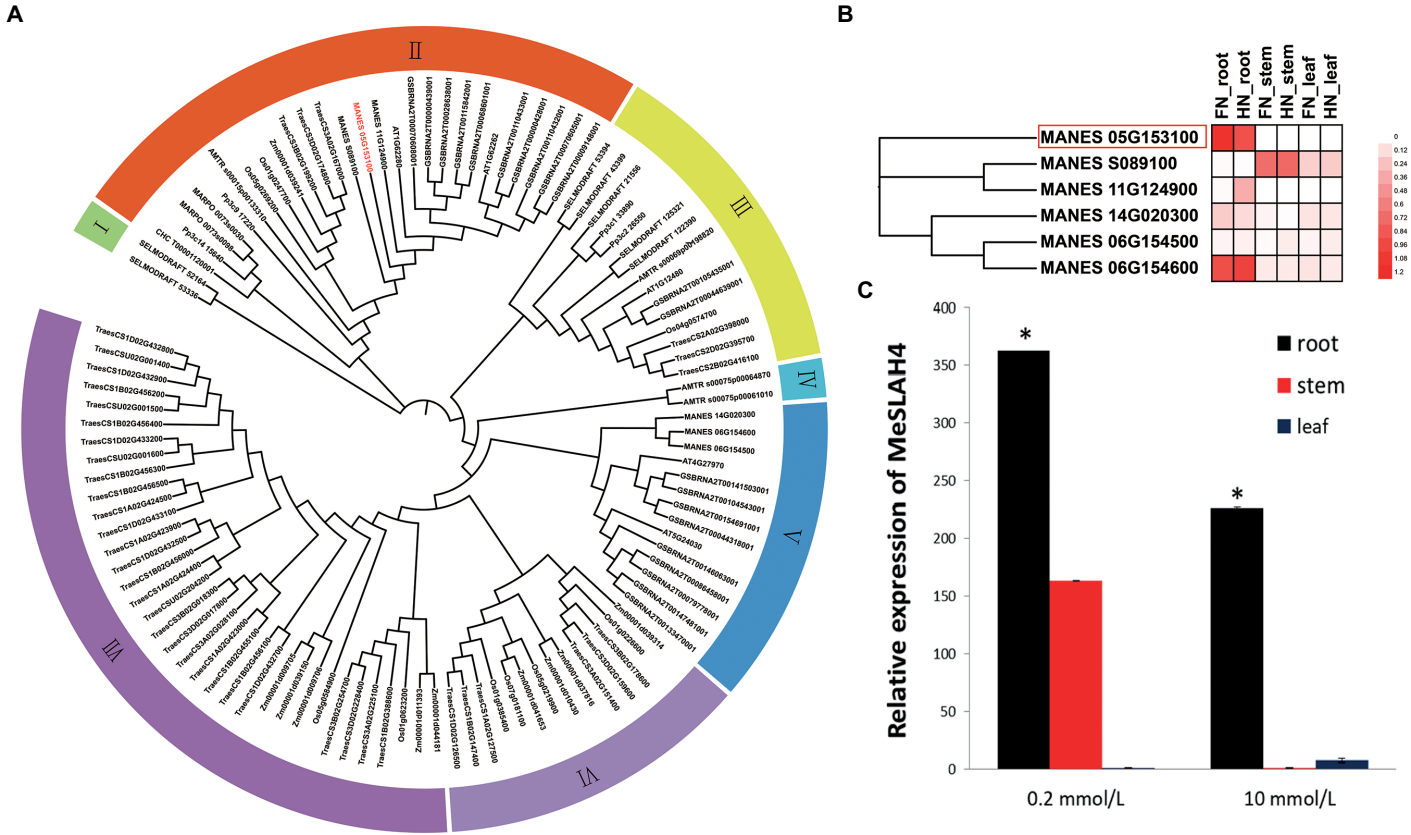
Phylogenetic relationship and expression analysis of *SLAH* genes. **(A)** Phylogenetic tree of SLAH proteins constructed using ML method with 1,000 bootstrap replications in different species. The prefixes CHC, Pp3c, MARPO, AMTR, Os, Zm, Traes, MANES, AT, GSBRNA, and SELMODRAFT represent *Chondrus crispus*, *Physcornitrella patens*, *Marchantia polymorpha*, *Amborella trichopoda*, *Oryza sativa*, *Zea mays*, *Triticum aestivum*, *Manihot esculenta*, *Arabidopsis thaliana*, *Brassica napus*, and *Selaginella moellendorffii*. **(B)** Expression profiles of *SLAH* genes in the root, stem, and leaf of cassava under different nitrogen conditions. FN (0 mmol/l of nitrate nitrogen), and HN (10 mmol/l of nitrate nitrogen). **(C)** Relative expression of the *MeSLAH4* gene in cassava. The relative expression levels were obtained by normalization with the *MeActin* gene. The error bars indicate standard deviations, and the data are shown as mean values ± SD, while ^*^ represents significant differences at *p* < 0.05 on one-way ANOVA analysis.

In the present study, a close phylogenetic relationship of entire *SLAH* genes in cassava with previously reported *SLAH* genes in another species was detected (Figure 1A) by analyzing with a Hidden Markov Model (HMM) profile search along with a conserved model (SLAC1, PF03595) for the SLAH proteins. The phylogenetic tree displayed seven clades (I to VII), and a very close relationship among the six *SLAH* genes in the cassava genome, five *SLAH* genes in *Arabidopsis*, and nine *SLAH* genes in rice were identified. However, two *SLAC1* gene homologues (*Os05g0269200* and *Os01g0247700*) in rice and two *SLAC1* homologues (*AT1G62280*, *SLAH1*; and *AT1G62262*, *SLAH4*) in *Arabidopsis* were detected in the same clade (Clade II) as the *MeSLAH4* (*MANES05G153100*) gene. Moreover, the MeSLAH4 protein demonstrated about 49.40% similarity with the Os05g0269200 amino acid sequence (Supplementary Table 1; Supplementary Figure 1). Besides, the phylogenetic tree using IQ-TREE of SLAH protein also revealed an evolutionary relationship with other species, including *Chondrus crispus* (Carrageen Irish moss), *Physcornitrella patens* (Bryophyta), *Marchantia polymorpha* (Liverwort), *Amborella trichopoda*, *Z. mays* (Corn), *T. aestivum* (Wheat), *B. napus* (Rapeseed), and *Selaginella moellendorffii* (Spikemoss).

RNA-seq data which are available on the database represents diverse expression pattern of *SLAH* genes in cassava, and the *SLAH* genes are expressed differentially in different tissues of cassava under different nitrogen conditions (Figure 1B). In particular, the *MeSLAH4* (*MANES_05G153100*) gene is highly expressed in the root under free nitrate concentration (FN, 0 mmol/l, around 1.2-fold) as well as at high nitrate concentration (HN, 10 mm/l, approximately 1.08-fold). Another *MeSLAH1* homologue 3 (*MANES_06G154600*) is also expressed in the roots of cassava but a little bit lower (FN, about 1.08-fold, and HN, roughly 0.96-fold) than the *MeSLAH4* gene.

The relative expression pattern of the *MeSLAH4* gene in different tissues of cassava revealed variations in expression levels at various concentrations of nitrate levels (Figure 1C). The transcript accumulation patterns that were analyzed in roots, stems, and leaves indicated that the *MeSLAH4* gene was mainly expressed in the root. A significantly higher expression (about 370-fold) was observed in the root under a lower concentration of nitrate (0.2 mmol/l) treatment (Figure 1C). Thus, the *MeSLAH4* gene exhibited significantly higher relative gene expression levels at lower nitrate concentrations in the root, indicating a potential role in enhancing nitrogen use in plants.

As the root is the principal organ for nutrient uptake in plants, the up-regulation of the *MeSLAH4* gene could correlate the relationships among different nitrate concentrations with plant growth and development.

### Chromosome Localization of *SLAH* Genes and Analysis of the Promoter Regions of *SLAH* Genes With *cis*-Acting Elements

**Figure 2:**
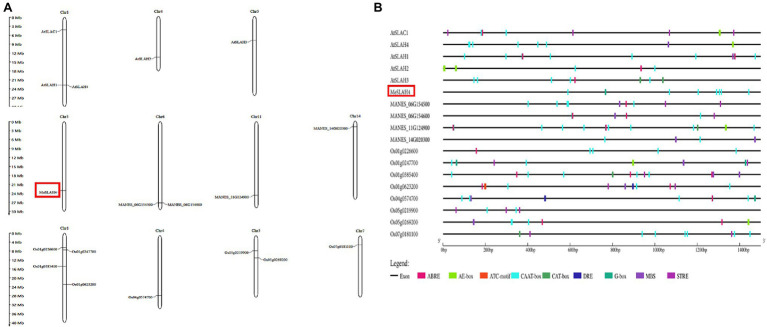
Chromosomal localization and *cis*-elements analysis. **(A)** Chromosome localization of *SLAH* genes in *Arabidopsis thaliana* (At), *Manihot esculenta* (MANES), and *Oryza sativa* (Os). The approximate positions of the *SLAH* genes are presented on the respective chromosome size. **(B)** *Cis*-elements in the promoter regions (2.0-kb upstream regions) of the *SLAH* genes in *Arabidopsis*, cassava, and rice. Different colors represent various types of *cis*-elements, including core promoter elements, light responsive, phytohormone responsive, abiotic stress responsive and others related to growth. ABRE (ABA-responsive elements); E-box (enhancer box); CCAAT box/CAAT box/CAT box (GGCCAATCT consensus sequence); DRE (DNA Replication-related Element); G-box (G - box sequence, CACGTG); MBS (multichain binding site); STRE (stress-responsive elements).

The *SLAH* genes are distributed on four chromosomes in cassava (chromosomes 5, 6, 11, and chromosome 14). In cassava, one *SLAH* gene, which has been identified as *MeSLAH4* genes, was present on chromosome 5, two *SLAH* genes were located on chromosomes 6, one gene on chromosome 11, and one gene on chromosome 14 (Figure 2A). The *SLAH* genes in *Arabidopsis* are detected on three chromosomes (three genes on chromosome 1, one gene on chromosome 4, and one gene on chromosome 5). In rice, four *SLAH* genes were found on chromosome 1, one gene on chromosome 4, two genes on chromosome 5, and one gene on chromosome 7 (Figure 2A).

Analysis of the upstream promoter region of *SLAH* genes represented transcriptional regulation mechanisms. About 2 kb upstream of the initiation codon of *SLAH* genes of *Arabidopsis*, cassava, and rice were obtained and submitted to the Plant CARE database for investigating *cis*-regulatory elements. A total of 9 different *cis*-elements associated with light responsiveness, stress responsiveness, phytohormone responsiveness and growth regulation have been identified in upstream regions of *SLAH* genes (Figure 2B).

A linear line has been constructed to present regulatory elements in each corresponding gene (Figure 2B). These results indicate that complex regulatory networks may be implicated in the transcriptional regulation of *SLAH* genes in different plants. *Cis*-regulatory elements, CAAT-box was commonly shared by all *SLAH* genes. G-box elements responding to light existed in the 2-kb upstream region of *SLAH* genes. Most *SLAH* genes contain ABRE elements (ABA responsive), but they are absent in the cassava *MeSLAH4* gene which suggested that this gene might not involve in regulation and physiological responses of various processes, including stomatal closure, seed and bud dormancy. Moreover, *SLAH* genes harbored drought responsive *cis*-elements (DRE) that were not present in the *MeSLAH4* gene. The *MeSLAH4* gene contains several copies of the CAAT-box and a copy of the G-box, indicating a higher transcription rate with sufficient quantities of suitable binding sites for several transcription factors as well as a highly conserved sequence for evolutionary process and epigenetic regulation.

### Subcellular Localization of MeSLAH4 Protein

**Figure 3:**
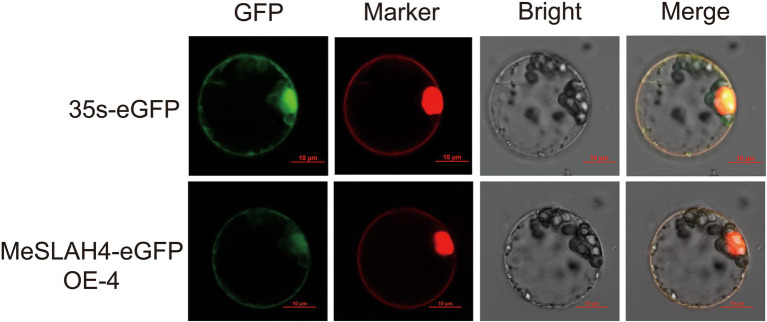
Subcellular location of MeSLAH4 proteins. MeSLAH4-eGFP or 35 s-eGFP driven by the 35S promoter were transiently expressed in rice mesophyll protoplasts. Green signals indicate eGFP and red signals represent mCherry fluorescence. The merged images include the green fluorescence channel (first panels) and the chloroplast autofluorescence channel (second panels). The corresponding bright field images are shown on the right. Bar = 10 μm.

The transiently expressed fusion protein driven by the 35S promoter through protoplast transformation of the *MeSLAH4* gene represented a clear subcellular localization in rice (Figure 3). The green fluorescent signal of eGFP, which represented a negative control, was observed in the cytoplasm. However, the signal of the MeSLAH4-eGFP fusion protein, which coincides with the red fluorescent signal of plasma membrane-localized protein, was detected in the plasma membrane and in the nucleus. The protein localization was further confirmed by the protoplast transformation, which indicated that MeSLAH4 proteins are localized in the plasma membrane and in the nucleus. The microscopic visualization exhibited that the green fluorescence was distributed throughout the whole cell when the control (empty) vector was used. The green fluorescence was exclusively detected on the plasma membrane and nucleus by confocal microscopy when the vectors contained MeSLAH4 (Figure 3). These results indicate that the *MeSLAH4* gene may be involved in other functions in the plants.

### Influence of *MeSLAH4* Overexpression on Morpho-Physiological Traits in Transgenic Rice

**Figure 4:**
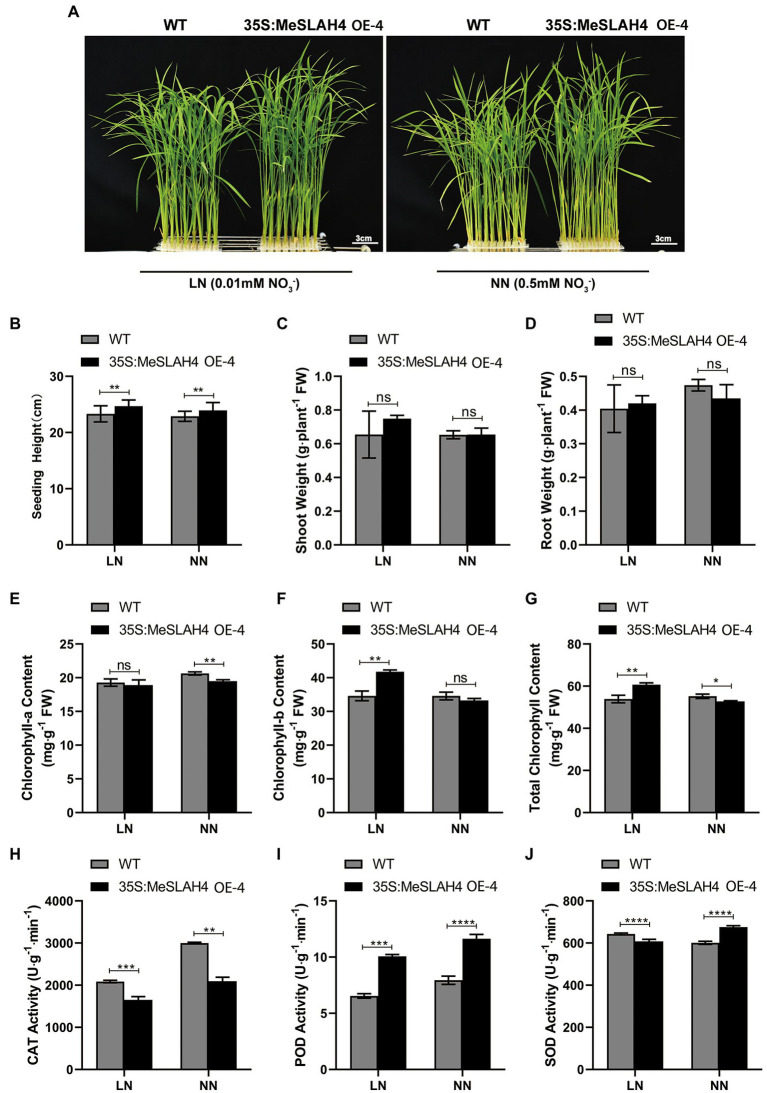
Morpho-physiological traits of wild type and OE lines under different nitrogen conditions. **(A)** Phenotypes of wild type (WT) and MeSLAH4-OElines (35S:MeSLAH4) grown in hydroponic medium with different nitrate concentrations for 14 days. Bar = 3.0 cm. **(B)** Plant height (cm). **(C)** Shoot weight (g plant^−1^ FW). **(D)** Root weight (g plant^−1^ FW). **(E)** Chlorophyll-a content (mg. g^−1^ FW). **(F)** Chlorophyll-b content (mg. g^−1^ FW). **(G)** Total chlorophyll content (mg. g^−1^ FW). **(H)** CAT activity (U.g^−1^.min^−1^). **(I)** POD activity (U.g^−1^.min^−1^). **(J)** SOD activity of seedings (U.g^−1^.min^−1^). The letters “ns” indicate non-significant differences while ^*^, ^**^, ^***^, and ^****^ represent significant differences at *p* < 0.05, *p* < 0.01, *p* < 0.001, *p* < 0.0001, respectively on one-way ANOVA analysis.

In the current experiment, the plant phenotype exhibited higher overall growth in OE lines under both nitrated concentrations (0.5 mm NH_4_NO_3_, NN, and 0.01 mm NH_4_NO_3_, LN) compared to the WT (Figure 4A). Plant height was significantly different (*p* < 0.01) under both nitrated concentrations (NN and LN) in both WT and OE lines, but they demonstrated higher plant height at LN compared to NN (Figure 4B). The shoot and root weight exhibited non-significant differences in both WT and OE lines under both nitrate concentrations (LN and NN; Figures 4C,D). However, shoot weight was higher (0.75 g. plant^−1^ FW) in OE lines at LN compared to both lines (WT and OE) in NN condition. Conversely, the root weight was higher in the WT (4.8 g. plant^−1^ FW) under NN than under LN. These results point out that the overexpression of the *MeSLAH4* gene enhances aboveground biomass (plant height and shoot weight) but decreases the lower ground parts (root weights) under low nitrate conditions.

The chlorophyll-a content was non-significantly different at LN but significantly different (*p* < 0.05) under NN while it was higher at NN condition (Figure 4E). Conversely, the chlorophyll-b content was significantly different (*p* < 0.05) under LN but a non-significant difference was observed under NN, while the OE lines showed higher chlorophyll-b content compared with WT and OE lines under LN conditions (Figure 4F). The OE lines showed higher total chlorophyll content under LN conditions, which also demonstrated significant differences (*p* < 0.05) compared to the WT (Figure 4G). Higher chlorophyll content in OE lines under LN indicates the increasing nitrogen use efficiency and higher conversion of photosynthesis by the plants under low nitrate concentration.

The CAT activity was significantly different under the LN (*p* < 0.01) and NN (*p* < 0.001) conditions, but both lines (WT and OE) demonstrated higher activity under the NN condition, where WT had more activity than OE lines (Figure 4H). The POD activity of the OE line was higher than wild type (WT) under both (LN and NN) conditions, but it was highly significant (*p* < 0.0001) under the NN condition (Figure 4I). The SOD activity was complicated because the WT demonstrated higher SOD activity (650 U.g^−1^min^−1^) under the LN condition while the OE lines exhibited higher activity (700 U.g^−1^min^−1^) under the NN condition (Figure 4J). Lower CAT and SOD activity of OE lines in LN conditions indicates higher photosynthetic and stress-responsive activities, while the activity of POD in OE lines under both (LN and NN) conditions demonstrates a higher ability to scavenge hydrogen peroxide under prolonged nitrated conditions.

### Effects of *MeSLAH4* Overexpression on Grain Morpho-Physiological Traits in Transgenic Rice

**Figure 5:**
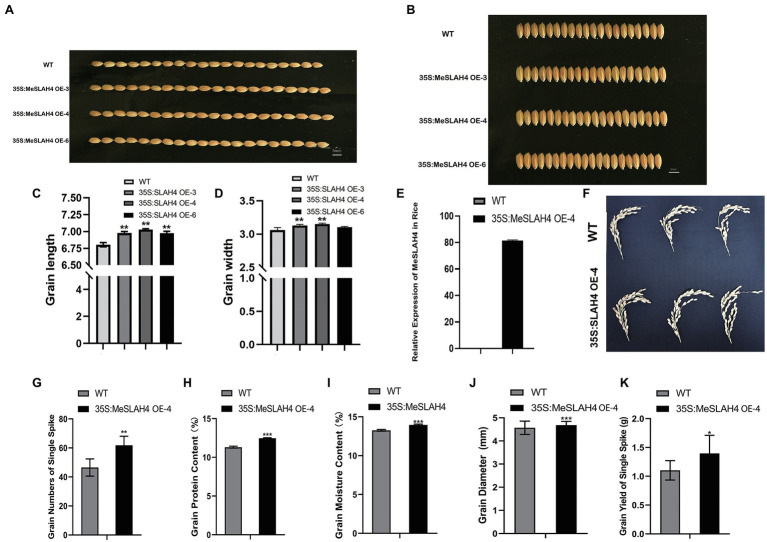
Morpho-physiological traits of grains and relative expression of 35S:MeSLAH4 gene in the experimental lines. **(A,C)** grain length (mm). **(B,D)** grain breadth (mm). Bar = 5.0 mm. **(E)** Relative expression of the 35S:MeSLAH4OE4 line related to *OsActin* gene. **(F)** Panicle morphology. **(G)** grain numbers on a spike. **(H)** Grain protein content (%). **(I)** Grain moisture content (%). **(J)** Grain diameter (mm). **(K)** Grain yield of a spike (g). The symbols ^*^, ^**^, and ^***^ represent significant differences at *p* < 0.05, *p* < 0.01, *p* < 0.001, respectively, on one-way ANOVA analysis.

In the field trial, the grain length (Figures 5A,C) and breadth (Figures 5B,D) of transgenic rice were increased significantly relative to the wild type, and the highest increment was observed in the 35S:MeSLAH4 OE-4 line. The relative gene expression of this line (35S,MeSLAH4 OE-4) was higher (about 80-fold, Figure 5E) and the panicle morphology (Figure 5F) was better than the wild type, hence the OE-4 lines have been selected for all other experiments. The grain numbers of a single spike (around 60) in the OE lines have been increased (Figure 5G). These results indicate that the OE lines have up-regulated *MeSLAH4* gene expression, which has facilitated higher assimilation rates and more storage in the grain compared to the wild types.

As shown in Figure 5, all other traits of the OE line, including grain protein content (12%), grain moisture content (14%), grain diameter (4.8 mm), and grain yield of a single spike (1.48 g), were higher compared to the WT. Thus, the results of this experiment demonstrated that overexpression of the *MeSLAH4* gene increases protein content with higher grain expansion and yield in rice.

### Effects of *MeSLAH4* Overexpression on Root Morphological Traits in Transgenic Rice

**Figure 6:**
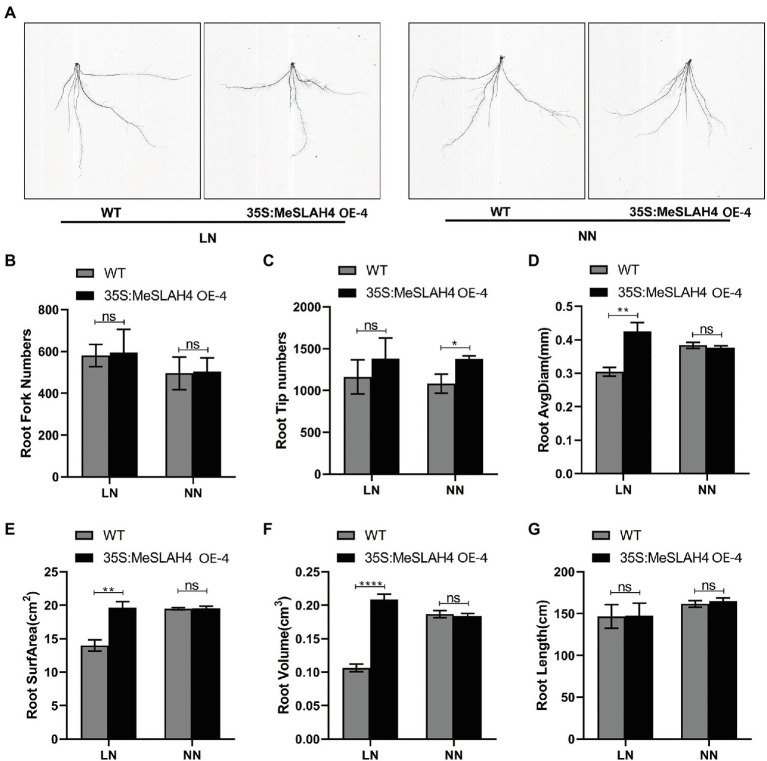
Root phenotypes of the wild type and OE line under different nitrate conditions. **(A)** Phenotypes of wild type (WT), and MeSLAH4-OE line (35S:MeSLAH4) grown in hydroponic medium treated with LN (0.01 mm ${\mathrm{NO}}_{3}^{-}$ ) and NN (0.50 mm ${\mathrm{NO}}_{3}^{-}$ ) for 14 days. **(B)** Root fork numbers (number of branches). **(C)** Root tip numbers. **(D)** Root average diagram (mm). **(E)** Root surface area (cm^2^). **(F)** Root volume (cm^3^). **(G)** Root length (cm) of seedings. The letters “ns” indicate non-significant differences while ^*^, ^**^, ^***^, and ^****^ represent significant differences at *p* < 0.05, *p* < 0.01, *p* < 0.001, *p* < 0.0001, respectively on one-way ANOVA analysis.

The root system indices (phenotype of roots) demonstrated changes in terms of size and shapes in the wild type (WT) and overexpression (OE) lines under different nitrate conditions (Figure 6A). The root fork numbers (number of branches) and root tip numbers were increased (580 and 490, respectively) under the LN condition, and the increment was higher in the OE line compared to the WT line (Figures 6B,C). These results indicate that a lower nitrate concentration facilitated the formation of a higher root number. The root average diagram of WT was increased in NN, but it was increased higher in OE under the LN condition compared to WT, as well as both (WT and OE) under the NN condition (Figure 6D). Similar types of expansions were observed in the OE line for root surface area (22.0 cm^2^, Figure 6E) and root volume (22.0 cm^3^, Figure 6F). However, root length was higher in the OE line (160 cm) under NN conditions, and it demonstrated non-significant changes compared to the WT (Figure 6G). Enlargement and expansion of root average diagram, root surface area, and root volume indicate that limited nitrate concentration does not inhibit root growth but rather allows it to optimize for absorption of more nutrient resources.

### Nitrogen Accumulation in Rice Lines

**Figure 7:**
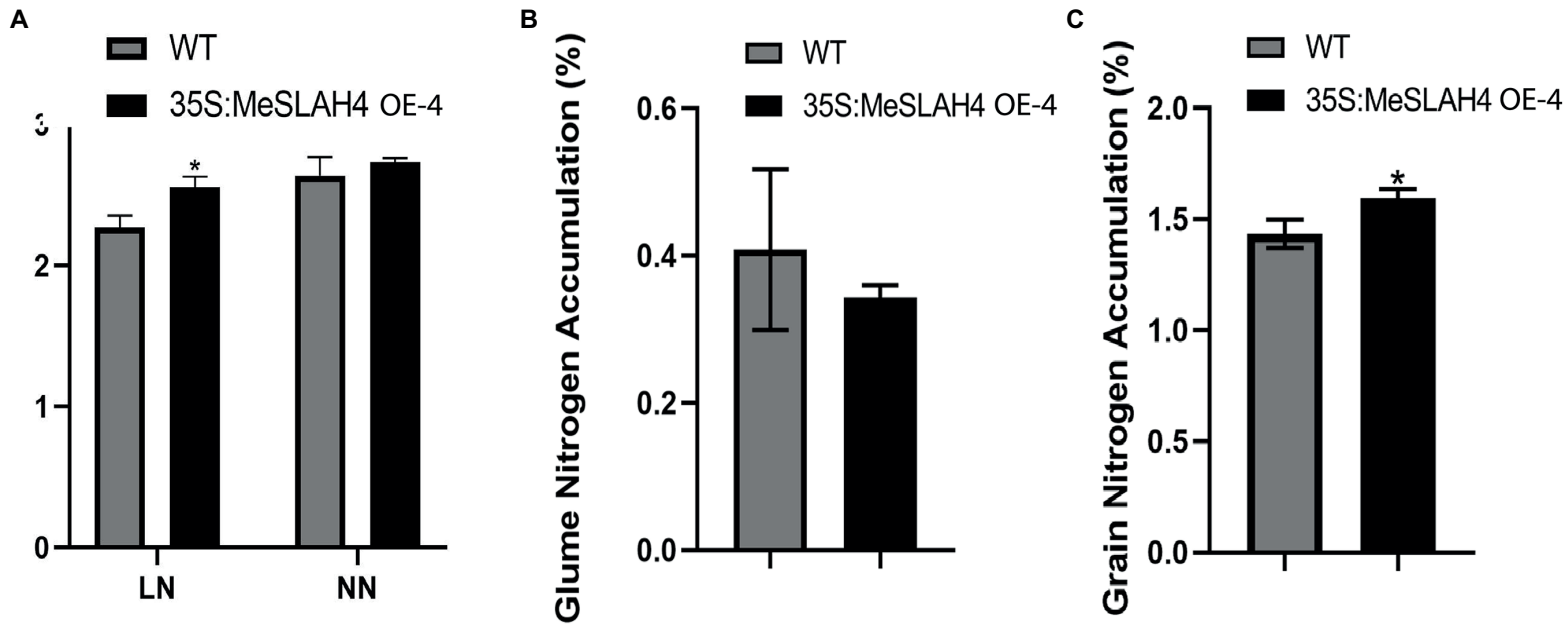
Nitrogen accumulation in whole plants, grain, and glume in rice. **(A)** Nitrogen accumulation in whole rice plants. LN, 0.01 mm NH_4_NO_3_; NN, 0.5 mm NH_4_NO_3_. **(B)** Nitrogen accumulation (%) in glume. **(C)** Nitrogen accumulation (%) in grain. The error bars indicate standard deviations and the data are shown as mean values ± SD, while ^*^ represents significant differences at *p* < 0.05 on one-way ANOVA analysis.

The nitrate accumulation in the transgenic whole plants (35S:MeSLAH4) was higher under both the low nitrate (LN) and normal nitrate (NN) conditions compared to their wild type. However, there was a significant difference in the nitrate accumulation in whole plants at the low nitrate concentration (Figure 7A). During organ or tissue-specific nitrate accumulation analysis, rice grain exhibited significantly higher nitrate accumulation in the transgenic lines compared to the wild type lines (Figure 7B). Conversely, transgenic rice lines demonstrated lower nitrate accumulation in the glume (Figure 7C).

These results showed that overexpression of the *MeSLAH4* gene led to more nitrate accumulation by whole plants, more storage in the grain, but lower translocations to the glume in rice.

## Discussion

Nitrogen (N) is one of the most important micronutrients required for plant growth and development, and hence, plants have evolved different strategies, sophisticated mechanisms, and adaption processes depending on soil N availability and distribution. Among the four gene families involved in the nitrate transport system, the *SLAC*/*SLAH* members play an important role in anion transport, stress signaling, growth and development, and hormonal response (Vahisalu et al., 2008; Nan et al., 2021). A total of five *SLAC*/*SLAH* genes were identified in *Arabidopsis* (Vahisalu et al., 2008), 23 genes in *B. napus* (Nan et al., 2021), and 9 genes in rice (Kusumi et al., 2012; Sun et al., 2016). In this study, six *SLAH* genes were identified in cassava, and these genes showed close phylogenetic relationships with other organisms (Figure 1A). These *SLAH* genes are expressed differentially in different tissues of cassava under varying nitrogen concentrations. Predominantly, the *MeSLAH4* (*MANES_05G153100*) gene was highly expressed in the root under free nitrate concentration (FN) as well as at high nitrate concentration (HN; Figure 1B), indicating a potential role in enhancing nitrogen use in plants. Since the root is the principal part for nutrient uptake in plants, the overexpression of the *MeSLAH4* gene has been tested in rice, which could correlate the relationships between different nitrate concentrations with plant growth and development parameters. In *Arabidopsis*, the *SLAH3* (*SLAC1* homologue 3) gene closely related to the *SLAC1* gene showed an overlapping function with *SLAC1* in guard cells (Negi et al., 2008; Geiger et al., 2011). The expression of the *SLAH3* gene was also detected in guard cells, albeit at much lower levels than the expression of *SLAC1* (Geiger et al., 2011; Zheng et al., 2015). High expression levels of the *SLAH3* gene were observed in roots and exhibited stronger selectivity for nitrate over chloride compared to *SLAC1* (Geiger et al., 2009; Lee et al., 2009), and therefore, it was considered a nitrate efflux channel. Although the *SLAH2* gene, which is the closest homolog of the *SLAH3* gene, is also expressed in root vascular tissues, it did not show any related phenotype under the same conditions as the *SLAH3* gene, indicating non-overlapping function (Zheng et al., 2015). In *Arabidopsis*, *SLAH1* and *SLAH4* genes share similar duplicates, and both are members of a clade that predates seed plants. However, similar to *SLAH3*, the *SLAH4* gene is also expressed in roots, and shows relatively stronger expression near the root tip (Zheng et al., 2015).

In this study, the cassava *MeSLAH4* gene was identified on chromosome 5 (Figure 2A) and localized in the plasma membrane and nucleus (Figure 3). The localization of the *MeSLAH4* protein in the plasma membrane and nucleus indicated that MeSLAH4 protein may be involved in other cellular functions. Previously, confocal microscopy observations pointed out that BnSLAH1-1, BnSLAH3-2, and BnSLAH3-3 were localized on the plasma membrane the same as in *Arabidopsis* and pear (Chen et al., 2019a; Nan et al., 2021).

Analysis of the upstream promoter region of *SLAH* genes (Figure 2B) showed that the *cis*-regulatory elements, CAAT-box, were commonly shared by all *SLAH* genes, and most *SLAH* genes contained ABRE elements (ABA-responsive) and drought-responsive *cis*-elements (DRE), which are absent in the cassava *MeSLAH4* gene suggested that this gene might not be involved in regulation and physiological responses of various processes, including stomatal closure, seed, bud dormancy, and stresses (Gómez-Porras et al., 2007). Besides, the *MeSLAH4* gene was observed to contain several copies of the CAAT-box and a copy of the G-box, indicating a higher transcription rate with sufficient quantities of suitable binding sites for several transcription factors. The CAAT box is generally located approximately 80 bp upstream of the transcription start site (TSS) and significantly influences gene expression efficiency (Biłas et al., 2016). In addition, the presence of the highly conserved G-box motif (CACGTG) indicated frequent binding with the basic helix–loop–helix (bHLH) and basic Leu zipper (bZIP) TF families (Ezer et al., 2017). In *B. napus*, promoter analysis showed the presence of different kinds of *cis*-elements involved in the light response, phytohormone response, drought response, low temperature response, and growth regulation. It was assumed that the *BnSLAC*/*SLAH* may function in the abiotic stress tolerance, and growth regulation (Nan et al., 2021).

A total of 10 motifs (motifs 1 to 10) were identified in the Arabidopsis, rice, and cassava *SLAH* genes (Supplementary Figures 2, 3), while the *MeSLAH4* gene in the cassava contains 5 motifs (motifs 1, 2, 4, 5, and motif 8), which might represent the conserved functional motif of this gene. In previous experiments on conserved motif analysis, it was suggested that the presence of motifs 1, 3, 4, 8, and 10 indicated a conserved functional motif in the *SLAC*/*SLAH* gene family of Rosaceae (Chen et al., 2019a). However, the *BnSLAH3* subfamily was found to contain motifs 1 to 10, while *BnSLAH2* contained motifs 1, 5, and 7. In the same experiment, motifs 1 to 7 were found to be widely distributed in the *BnSLAH1*, *BnSLAH4*, and *BnSLAC1* subfamilies (Nan et al., 2021). These conserved motifs were considered to have functional or structural roles in active proteins, indicating functional diversity during growth and development in plants (Nan et al., 2021).

In the current experiment, plant height was significantly different (*p* < 0.01) under both nitrated concentrations (LN and NN) in both WT and OE lines, but they demonstrated higher plant height at LN compared to NN (Figure 4B). The shoot weight was higher in OE lines at LN compared to both lines (WT and OE) in NN condition. Conversely, the root weight was higher in the WT under NN than under LN. Thus, overexpression of the *MeSLAH4* gene enhances aboveground biomass (plant height and shoot weight) but decreases the lower ground parts (root weights) under low nitrate conditions. In an earlier experiment, overexpression of the *OsNLP4* gene significantly increased N uptake and assimilation in rice, thus enhancing plant growth, grain yield and NUE compared with the wild type under all N conditions (Wu et al., 2021). The OE lines showed higher total chlorophyll content compared to the WT (Figure 4G) under LN conditions with significant differences (*p* < 0.05), indicating higher nitrogen use efficiency with higher conversion of photosynthesis under low nitrate concentration. Previously, transgenic plants (overexpression of *OsGS1;1* and *OsGS2* genes) exhibited higher chlorophyll fluorescence under stress (drought and salinity) compared to control rice plants, which indicated that the transgenic lines had enhanced protection of the photosynthetic machinery, leading to improved post-stress recovery (James et al., 2018). Lower CAT (Figure 4H) activity and less SOD (Figure 4J) activity of OE lines in the LN condition indicated higher stress-responsive activities, while higher activity of POD (Figure 4I) in OE lines under both (LN and NN) conditions demonstrated a higher ability to scavenge hydrogen peroxide under prolonged nitrated conditions. The plants that were deficient in CAT indicated an association with photorespiratory H_2_O_2_ accumulation and downstream oxidative signaling (Vandenabeele et al., 2004). The SOD enzyme catalyzes the dismutation of the superoxide anion ( $O_{2}^{•-}$ ) into hydrogen peroxide and molecular oxygen, which play the most important roles in protecting against oxidative stress as well as in the survival of plants under stressful conditions (Gill and Tuteja, 2010). The activity of POD is increased under decreased CAT activity to compensate for the lack of H_2_O_2_ scavenging capacity in rice under stress conditions (Wang et al., 2019).

The root fork numbers (number of branches) and root tip numbers were increased under the LN condition, and the increment was higher in the OE line compared to the WT line (Figures 6B,C). Enlargement and expansion of root average diagram (Figure 6D), root surface area (Figure 6E), and root volume (Figure 6F) in OE lines indicating optimize condition for higher absorption of nutrients. Former researchers discovered that *BnSLAH3-2*, *BnSLAH3-3*, and *BnSLAH3-4* were up-regulated in roots 12 h after low nitrate treatment (0.19 mm), indicating that the *BnSLAH3* genes could respond quickly to low nitrate stress and may promote nitrate uptake and transport in rapeseed roots. Conversely, a high concentration (64 mm) of nitrate was detected to induce expression of *SLAC*/*SLAH* genes in pear, which indicated that gene expression varies depending on species, nitrate concentration, and treatment time (Chen et al., 2019a; Nan et al., 2021).

The nitrate accumulation in the transgenic plants (35S,MeSLAH4) was higher under both nitrate (LN and NN) conditions compared to their wild type, but it was significantly different at the low nitrate concentration (Figure 7A). However, higher nitrate accumulation led to more storage in the grain but lower translocations to the glume in rice. In the present experiments, other traits, including grain numbers of a single spike, grain protein content, grain moisture content, grain diameter, and grain yield of a single spike of the OE line, were higher compared to the WT (Figure 5). It is well known that crop yield is closely related to N utilization, and it mainly depends on nitrogen absorption by plants before flowering and nitrogen remobilization during seed maturation (Kichey et al., 2007; Masclaux-Daubresse et al., 2008). Current research reveals that overexpression of the *MeSLAH4* gene significantly enhances grain size as well as nitrate influx in OE-lines compared to the wild type. Hence, the *MeSLAH4* gene might play an important role in the process of nitrogen transport and nitrogen utilization efficiency, which could be useful in developing high-yielding crop varieties. In addition, this study found that *MeSLAH4* has great impacts on the biological function, regulatory mechanism of nitrate absorption and utilization, and enhanced performance of yield-related traits in rice.

## Conclusion

Cassava is a short-day, durable, and easy-to-plant dicot plant with high adaptability and a huge yield that can obtain sufficient nitrogen from the soil without requiring excessive nitrogen fertilizer. For the improvement and production of nitrogen-efficient germplasm resources, it is crucial to identify the key genes involved in nitrogen-efficient utilization. However, it is well evident that the *SLAC*/*SLAH* genes play important roles in responses to nitrate transport, stress signaling, and growth and development in plants. Till date, detailed bioinformatic analyses of the *SLAC*/*SLAH* gene family in the cassava genome have not been reported completely. Only some identified gene information is available in the Phytozome and NCBI databases. The functional characterization and expression analysis of these genes remain to be elucidated. Hence, in this study, six *SLAC*/*SLAH* genes were identified in the cassava genomes, which demonstrated a close phylogenetic relationship with other organisms. The structural characteristics of the promoter region, gene expression analyses, motif and sequence logo comparisons, and chromosomal localizations with *Arabidopsis* and rice homologs have provided a suitable framework for analyzing the *SLAC*/*SLAH* genes in the cassava genome. Cassava *SLAH* genes, particularly the *MeSLAH4* gene, respond significantly to different concentrations of nitrate ions and are expressed highly in the roots and enhance grain dimension while increasing yield in rice. The *MeSLAH4* gene is identified on chromosome 5 and is localized in the plasma membrane and nucleus. The overexpression (OE) rice lines showed higher total chlorophyll content, increased root fork numbers (number of branches), and root tip numbers compared to the WT under low nitrate (LN) conditions. The findings of these studies revealed the potential of the *MeSLAH4* gene for use in high-yielding crop production, as well as laid the groundwork for future research into the other *SLAC*/*SLAH* genes found in the cassava genome.

## Data Availability Statement

The original contributions presented in the study are included in the article/Supplementary Material, further inquiries can be directed to the corresponding authors.

## Author Contributions

LS, XW, ZP, LJ, and LZ accomplished and finalized the experiment, performed data analysis, and prepared a draft of the manuscript. LS, LJ, JhY, JnY, and GL conducted experimental trails and collected data. LZ, XW, CW, and RZ constructed the transformation vector and produced transgenic plants. LS, SL, JhY, and YZ participated in morpho-physiological data measurements and prepared figures. XZ and WL provided guidance for the experimental design, analysis, and writing. ZP, XJ, XZ, WL, and ZZ designed, monitored, and validated the experimental procedures and corrected the final manuscript. All authors contributed to the article and approved the submitted version.

## Funding

This work was supported by the Hainan Provincial Natural Science Foundation of China (grant no. 2019RC303), the Major Science and Technology Plan of Hainan Province (grant no. ZDKJ2021012), the Advanced Scientific Program for the Returned Overseas Chinese Scholars, Henan Province (grant no. 30602724), the Henan Province Science and Technology Attack Project (grant no. 222102110465), the Special Fund for High-level Talent Research Team of Neijiang Normal University (grant no. RSC202102), and the State Key Laboratory for Managing Biotic and Chemical Treats to the Quality and Safety of Agro-products (grant nos. KF20200107 and KF202218).

## Conflict of Interest

The authors declare that the research was conducted in the absence of any commercial or financial relationships that could be construed as a potential conflict of interest.

## Publisher’s Note

All claims expressed in this article are solely those of the authors and do not necessarily represent those of their affiliated organizations, or those of the publisher, the editors and the reviewers. Any product that may be evaluated in this article, or claim that may be made by its manufacturer, is not guaranteed or endorsed by the publisher.

## Footnotes

^1^ http://www.arabidopsis.org/

^2^ https://rapdb.dna.affrc.go.jp/

^3^ http://phytozome.jgi.doe.gov/pz/portal.html

^4^ https://www.ncbi.nlm.nih.gov/geo/query/acc.cgi?acc

^5^ http://mg2c.iask.in/mg2c_v2.1/

^6^ http://gsds.cbi.pku.edu.cn/

^7^ http://meme.nbcr.net/meme/cgibin/meme.cgi

^8^ http://smart.emblheidelberg.de/

## Supplementary Material

Supplementary Figure 1 Amino acid alignment of MeSLAH4 protein in cassava, and Os05g0269200 protein in rice. Here, MANES (*Manihot esculenta*) MeSLAH4 amino acid sequence was the query sequence and it was aligned with Os05g0269200 amino acid sequence.

Supplementary Figure 2 Conserved motifs of *SLAH* genes. Here, MANES (*Manihot esculenta*), At (*Arabidopsis thaliana*), and Os (*Oryza sativa*) genes and their motifs are depicted. Various color represents different motifs. The lengths and positions of the colored blocks correspond to the lengths and positions of motifs in the individual protein sequences. The scale indicates the lengths of the proteins as well as the motifs.

Supplementary Figure 3 Sequence logos of the conserved motifs of *SLAH* genes. Over-represented motifs were identified using the MEME tool. The stack’s height indicates the level of sequence conservation. The heights of the residues within the stack indicate the relative frequencies of each residue at that position.

Supplementary Figure 4 Identification of transgenic rice lines. Lane 1–7 is a single transgenic strain of rice (35S:MeSLAH4OE-4), − WT control, + is MeSLAH4 plasmid.
