# Supplementary figures and images for: Cassava (Manihot esculenta) Slow Anion Channel (MeSLAH4) Gene Overexpression Enhances Nitrogen Assimilation, Growth, and Yield in Rice
Source: Front Plant Sci. 2022 Jun 27;13:932947. doi: 10.3389/fpls.2022.932947 (PMC9271942; doi:10.3389/fpls.2022.932947)

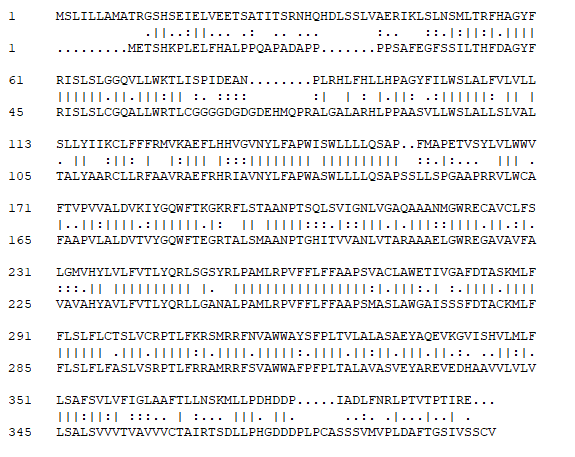

Supplement: Supplementary Figure 1 — Amino acid alignment of MeSLAH4 protein in cassava, and Os05g0269200 protein in rice. Here, MANES (Manihot esculenta) MeSLAH4 amino acid sequence was the query sequence and it was aligned with Os05g0269200 amino acid sequence. [file Image_1.png]

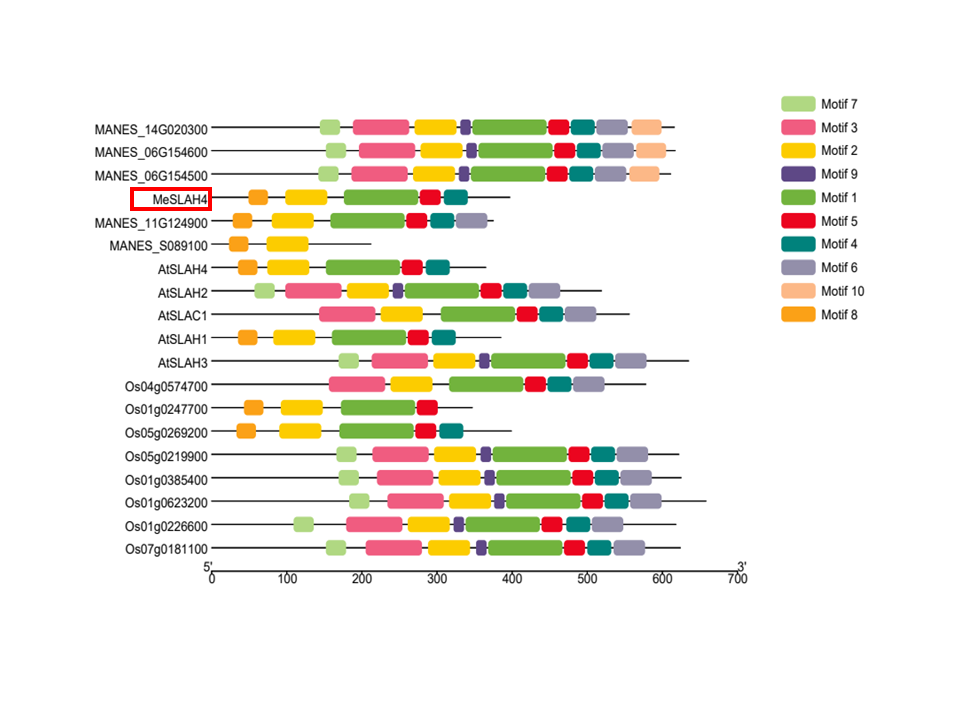

Supplement: Supplementary Figure 2 — Conserved motifs of SLAH genes. Here, MANES (Manihot esculenta), At (Arabidopsis thaliana), and Os (Oryza sativa) genes and their motifs are depicted. Various color represents different motifs. The lengths and positions of the colored blocks correspond to the lengths and positions of motifs in the individual protein sequences. The scale indicates the lengths of the proteins as well as the motifs. [file Image_2.png]

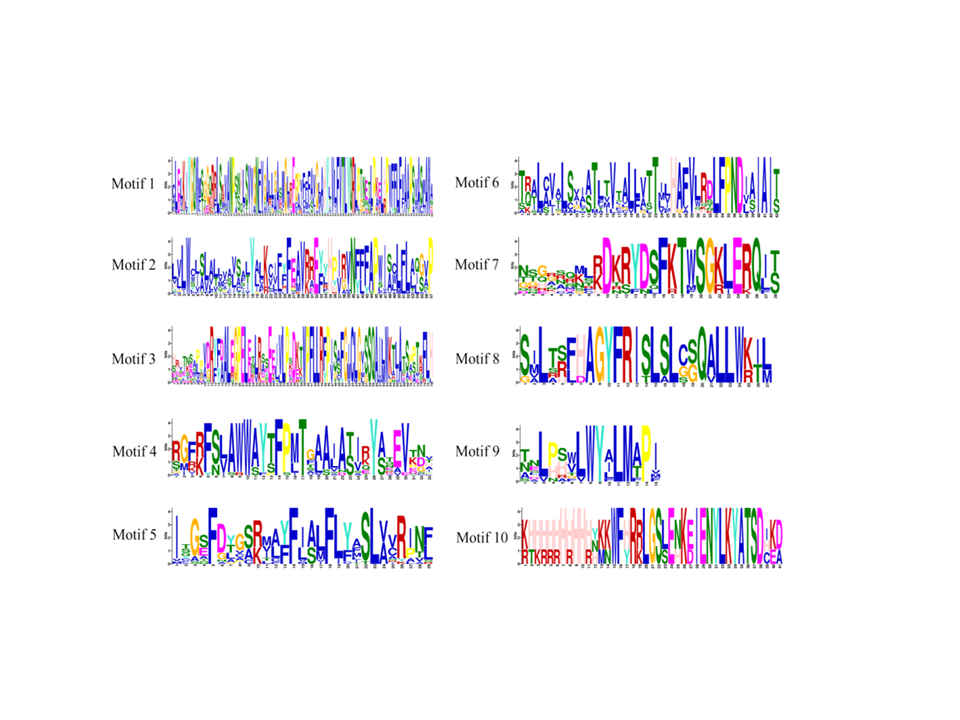

Supplement: Supplementary Figure 3 — Sequence logos of the conserved motifs of SLAH genes. Over-represented motifs were identified using the MEME tool. The stack’s height indicates the level of sequence conservation. The heights of the residues within the stack indicate the relative frequencies of each residue at that position. [file Image_3.png]

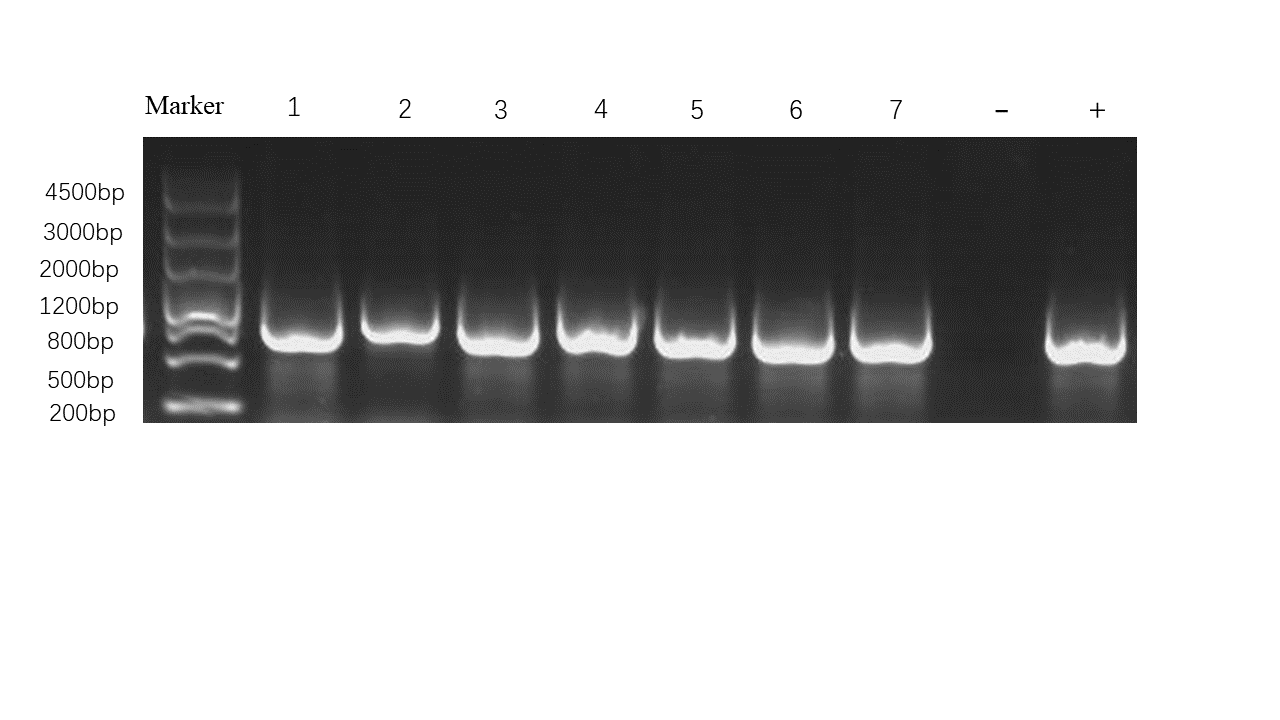

Supplement: Supplementary Figure 4 — Identification of transgenic rice lines. Lane 1–7 is a single transgenic strain of rice (35S:MeSLAH4OE-4), − WT control, + is MeSLAH4 plasmid. [file Image_4.png]
