# Supplementary material for: Cassava (Manihot esculenta) Slow Anion Channel (MeSLAH4) Gene Overexpression Enhances Nitrogen Assimilation, Growth, and Yield in Rice
Source: Front Plant Sci. 2022 Jun 27;13:932947. doi: 10.3389/fpls.2022.932947 (PMC9271942; doi:10.3389/fpls.2022.932947)
Supplement: Supplementary file 5 [file Data_Sheet_1.docx]

**Supplementary Table 1. Similarity percentage of protein sequences between MeSLAH4 and Rice SLAC/SLAH.**

| Gene ID | Protein sequence similarity percentage |
| --- | --- |
| Os05t0269200 | 49.40% |
| Os01t0247700 | 42.25% |
| Os01t0385400 | 28.94% |
| Os05t0219900 | 28.94% |
| Os04t0574700 | 24.57% |
| Os07t0181100 | 23.49% |
| Os01t0226600 | 22.70% |
| Os01t0623200 | 21.56% |
| Os05t0584900 | 9.28% |

**Supplementary Table 2. Primers used in this study.**

| Primer | Primer sequence (5′-3′) |
| --- | --- |
| Primers used for vector construction | |
| MeSLAH4-F | ggagaggacagggtacccgggATGTTAACGAGATTTCATGCAGGT |
| MeSLAH4-R | cttgctcaccatggtactagtTTCTCTAATTGTTGGTGTTACAGTTGG |
| Primers used for qRT-PCR | |
| MeSLAH4-F | CTCCAATCAGCACCTTTCA |
| MeSLAH4-R | GGTTTCCTATCACCGATAACT |
| Rice Actin-F | CAACACCCCTGCTATGTACG |
| Rice Actin-R | CATCACCAGAGTCCAACACAA |
| MeActin-F | GGCTCCTCTCAATCCTAAGG |
| MeActin-R | GTGTGGAAGAGCATACCCT |
